# Measuring Consumer Willingness to Pay to Reduce Health Risks of Contracting Dengue Fever

**DOI:** 10.3390/ijerph17051810

**Published:** 2020-03-10

**Authors:** Cheng-Te Lin, Yu-Sheng Huang, Lu-Wen Liao, Chung-Te Ting

**Affiliations:** 1 School of Business, Yulin Normal University, Yulin 537000, China; scsbte@gmail.com; 2 Department of Tourism, Food & Beverage Management, Chang Jung Christian University, Tainan City 71101, Taiwan; yshuang@mail.cjcu.edu.tw; 3 Department of Business Management, College of Intelligence, National Taichung University of Science and Technology, Taichung City 40401, Taiwan; lwliao@gm.nutc.edu.tw

**Keywords:** dengue fever, health risk, contingent valuation method, willingness to pay

## Abstract

Located in the subtropics, Taiwan is one of the major epidemic areas for dengue fever, with severe epidemics occurring in recent years. Dengue fever has become a serious health threat to Taiwan’s residents and a potentially serious economic cost to society. This study recruited 730 random participants and adopted the contingent valuation method to understand the factors influencing the populace’s willingness to pay (WTP) to reduce the health risk of dengue fever. The results show that high-income women with children and people with higher preventive perceptions and behavior are more willing to invest in preventive measures against dengue fever. In the evaluation of WTP for preventive treatment for health risks, each person was willing to pay on average NT$751 annually to lower psychological health risks, NT$793 annually to lower the risk of illness, and NT$1086 annually to lower the risk of death.

## 1. Introduction

Dengue fever is a viral infection primarily spread by *Aedes aegypti* mosquitoes. The infection causes a flu-like illness and can sometimes lead to potentially lethal complications [1]. The last decade has seen a dramatic increase in the incidence of dengue fever around the world, with half the global population at risk, especially in the Americas, Southeast Asia, and the Western Pacific Region [2]. Morin et al. [3] contended that climate factors such as warm temperatures, precipitation, and humidity are major drivers of the spread of dengue fever. As such, in times of abnormal climate and frequent population movement, the occurrence of dengue fever in human residential communities can lead to severe outbreaks, posing a serious threat to public health.

Dengue fever is prevalent across tropical and subtropical regions worldwide [1]. Taiwan straddles the Tropic of Cancer and has both tropical and subtropical climates. Environmental factors such as warm temperatures and rainfall promote the spread of dengue fever. Since the 20th century, Taiwan has had a dengue epidemic every year, often triggered by imported cases [4]. The hot and humid climate and the high population density in urban areas of cities in southern Taiwan make them major dengue hotspots [5]. Since Taiwan’s initial dengue epidemic in 1987–1988, there has been dengue outbreak almost every year in cities in southern Taiwan. In 2015, Tainan experienced the most severe dengue outbreak in history, with nearly 23,000 confirmed cases, demonstrating how dengue has already seriously affected the health and quality of life for residents of Taiwan and resulted in considerable psychological and physiological harm. Dengue epidemics not only pose risks to human health and life, but also markedly affect a country’s social security, economic development, tourism, and international image.

To lessen the extent of harm caused by dengue fever and safeguard the health and safety of its citizens, Taiwan’s government has adopted many dengue fever preventive measures, including epidemic surveillance, vector research, medical treatment, inspections, awareness campaigns, chemical spraying, and removal of breeding sources. However, the target mosquitoes have built up resistance to breeding source removal and environmental agents, and with the warmer climate extending their lifespan, these measures are no longer able to prevent epidemics from occurring. At present, no effective vaccines or antiviral agents exist to combat dengue fever [6]. Jose [7] suggested that the most effective preventive measures are to eliminate potential breeding grounds, such as containers or places that collect water, to stop vector mosquitoes from multiplying, and to avoid being bitten by vector mosquitoes. In the absence of effective antiviral drugs or available vaccines, vector mosquito control activities continue to be the primary method for preventing the spread of dengue [8].

Espinoza-Gomez et al. [9] contended that educational campaigns on dengue awareness, attitudes, and behavior are more effective than chemical spraying. Some researchers [8,10,11] have suggested using media exposure to reinforce community awareness and behavior. Gubler and Clark [12] suggested adopting disease prevention and mosquito control measures in communities because these can be cost effective and the most efficient interventions in the long term [8]. Public perception of the health risks and protective behaviors are critical for the prevention and control of diseases [13]. However, despite positive attitudes and perceptions regarding the adoption of preventive measures against dengue, lack of commitment in actually implementing prevention programs [14] can result in unsatisfactory results in overall dengue prevention. Beyond its psychological and physiological effects, dengue fever also affects the lives and safety of the public and indirectly influences the public’s perception of and responses to dengue prevention methods. Every person has a different perception of dengue fever, resulting in different perceived risks of a dengue epidemic.

Risk perception is the public’s impression of the likelihood of possible failure or negative results when purchasing or using products or services [15]. Lenz [16] divided risk perception into two categories, the occurrence of uncertain events (risk uncertainty) and the chance of loss as a result of the occurrence (risk of loss). This uncertainty about the future and the possibility of loss form the concept of risk. The public’s risk perception must be analyzed according to the public’s level of understanding of a danger, its consequences, and individual levels of risk-taking [17]. When the public lacks understanding about certain matters, their perception of its risk is higher; however, when something is understood, the risk is perceived to be lower [18].

Risk perception is a crucial aspect in many health behavior theories and is usually an intervening variable for changing health behaviors [19]. This is known as health risk perception. Drossaert et al. [20] stated that health risk perception is a person’s subjective assessment of the possibility of contracting or developing a particular disease or some other health problem. Anthonj et al. [21] argued that the public’s perception of health risks stimulates and shapes health-related behaviors and reduces risk exposure, for example, preventive health behaviors, such as adopting appropriate protective measures, not only depend on awareness of objective health risks but are also influenced by health beliefs and specific health perceptions [21,22]. When the public’s perception of health risks is successfully changed, it usually results in changes in health behaviors [23]. If the public perceives a health risk to be greater, they are more likely to engage in preventive behavior [24]. For example, an individual with a higher perception of the dengue health risk will proactively eliminate containers of stagnant water in the home environment to prevent the breeding of vector mosquitoes, reducing the rate of dengue infection. Conversely, individuals with a lower perception of the dengue health risk will have higher rates of dengue infection.

Perceptions of health risks may influence social welfare [19]. To draft more effective public policies regarding health risks that optimize social welfare, government agencies must understand the costs and benefits of lowering health risks. However, the costs and benefits of health risks cannot be monetarily valued using methods for appraising market goods; thus, the contingent valuation method (CVM) for nonmarket goods has been accepted as the appropriate method of assessing the public’s willingness to pay (WTP) for reducing health risks [25]. The public’s perception of health risks affects not only public health outcomes but also the WTP to reduce dengue epidemics. Some studies have assessed WTP to reduce rates of illness or death [26,27,28,29,30], but there has been no assessment of psychological health risks and WTP. Gunathilaka et al. [31] studied the impact of dengue fever on mental health in Sri Lanka, and pointed out that if people suffer from dengue fever, psychiatric manifestations may occur, such as depression, anxiety, and pain. To overcome this research gap, this study considered psychological health risks. Thus, this study divided the health risks of dengue fever prevention into three categories: psychological, illness, and death risks to assess respondents’ WTP for disease prevention at different levels of risk.

## 2. Materials and Methods

### 2.1. Theoretical Basis of the CVM

The CVM was first proposed in 1947 by Ciriacy Wantrup. It uses the technique of direct interviews to determine the value a person would pay (WTP) or accept (willingness to accept) for nonmarket goods. Surveys and experiments that adopt this method pose hypothetical questions to elicit the interviewees’ preferences and valuations [32]. The hypothetical questions do not aim to elicit the interviewees’ opinions and attitudes but rather the price an individual would be willing to pay or accept based on their valuation of the items under hypothetical conditions [33].

The basic CVM theoretical model is:

$$Max\;U\;=\;U\left(X,F\right),\;st.P_{X}\cdot X+P_{F}\cdot F\;=\;I,$$

where U = U(X,F) is the utility function of goods *X* and nonmarket goods *F*, $P_{X}$ is the price vector of X, and $P_{F}$ is the price vector of *F* (such as the cost of epidemic prevention), and I is the income. To maintain the original cost that utility level *U*^0^ is willing to pay, the utility function and expenditure function are, respectively:

$$U\left(F_{0},I_{0}\right)\;=\;U\left(F^{+},I_{0}\;-\;WTP\right),\;\mathrm{and}\;WTP\;=\;I\left(F^{+},U_{0}\right)\;-\;I_{0},$$

where WTP represents the cost that the public is willing to pay for effective dengue prevention and control. When the public accepts dengue prevention measures that can lower infection rates, their perception of health risks can be improved ($F_{0}$ is changed to $F^{+}$). Due to different individual traits and information sources among the public, there will be different WTP costs when receiving messages of effective dengue prevention; therefore, WTP is represented as:

$$WTP_{i}\;=\;f\left(x_{i}\right)\;+\;\varepsilon_{i},$$

where $WTP_{i}$ represents the public’s WTP, $\varepsilon_{i}$ is the residual and is consistent with the assumption $N\left(0,\sigma^{2}\right)$, and $x_{i}$ is the explanatory variable vector of the number i interviewee.

### 2.2. Dichotomous Choice Model

Bishop and Heberlein [34] introduced the closed-ended format for inquiring into the public’s WTP, otherwise known as the single-bounded dichotomous choice (S-BDC). The S-BDC inquiry format assigns a particular starting WTP (*B_i_*), and the interviewee expresses if they are willing to pay *B_i_* to improve the environment and quality of health; conversely, the interviewee can also express if they are unwilling to pay *B_i_*. As a result, the interviewee’s decision will lead to one of two possible responses—willing to pay or unwilling to pay—the probability of the derived combinations are π^Y^ and π^N^, respectively, as expressed in the following:

(1) $$\pi^{Y}\left(B_{i}\right)\;=\;\mathrm{Pr}\left\{{\mathrm{YES}\;\mathrm{to}\;B}_{i}\right\}\;=\;\mathrm{Pr}\left\{B_{i}\;\leq {\;\mathrm{WTP}}_{\max}\right\}\;=\;1\;-\;G\left(B_{i};\;\theta \right),$$

(2) $$\pi^{N}\left(B_{i}\right)\;=\;\mathrm{Pr}\left\{{\mathrm{NO}\;\mathrm{to}\;B}_{i}\right\}\;=\;\mathrm{Pr}\left\{B_{i}\;>{\;\mathrm{WTP}}_{\max}\right\}\;=\;G\left(B_{i};\;\theta \right),$$

where ${\mathrm{WTP}}_{\max}$ is the maximum value the interviewee is willing to pay, G(·) is the cumulative probability distribution, and θ  is the parameter vector.

Assuming interviewee (N) and assigned inquiry amount ($B_{i}^{s}$), the number i interviewee’s log-likelihood function is as follows:

(3) $${\mathrm{lnL}}^{s}\left(\theta \right)\;=\;\sum_{i=1}^{N}\left\{d_{i}^{Y}{\ln\pi }^{Y}\left(B_{i}^{s}\right)\;+{\;d}_{i}^{N}{\ln\pi }^{N}\left(B_{i}^{s}\right)\right\}\;=\;\sum_{i=1}^{N}\left\{d_{i}^{Y}\ln\left[1\;-\;G\left(B_{i};\;\theta \right)\right]\;+{\;d}_{i}^{N}\mathrm{lnG}\left(B_{i};\;\theta \right)\right\},$$

where the assigned amount $B_{i}^{s}$ is subject of the inquiry, if the interviewee is *willing* to pay, then $d_{i}^{Y}\;=\;1$ and $d_{i}^{N}\;=\;0$; conversely, if the interviewee is *unwilling*, then $d_{i}^{N\;}=\;1$ and $d_{i}^{Y}\;=\;0$.

The first-order equilibrium solution ($\frac{\partial {\mathrm{lnL}}^{s}\left({\hat{\theta }}^{s}\right)}{\partial \theta }\;=\;0)$ is taken from the log-likelihood function (${\mathrm{lnL}}^{s}\left({\hat{\theta }}^{s}\right)$) against the estimator (θ) to obtain the maximum approximate estimator $\left({\hat{\theta }}^{s}\right)$. The asymptotic variance–covariance matrix of ${\hat{\theta }}^{s}$ is expressed as follows:

(4) $$V^{s}\left({\hat{\theta }}^{s}\right)\;=\;{\left[-E\cdot \partial^{2}{\mathrm{lnL}}^{s}({\hat{\theta }}^{s})/\partial \theta \partial \;\theta \;\prime \right]}^{-1}\equiv I^{s}{\left({\hat{\theta }}^{s}\right)}^{-1},$$

where $I^{s}\left({\hat{\theta }}^{s}\right)$ represents the information matrix.

The relationship between the explanatory variable and WTP is represented as follows:

(5) $${\mathrm{WTP}}_{\max}\;={\;X}_{i}^{\text{’}}\theta \;+{\;\varepsilon }_{i},$$

where $X_{i}^{\text{’}}$ is the exogenous variable vector, θ is the parameter vector, and $\varepsilon_{i}$ is the residual vector.

Hanemann et al. [35] and Kanninen [36] developed the double-bounded dichotomous choice (D-BDC) method, which builds on the basic concept of S-BDC; after answering the first inquiry about their WTP (*B_i_*), the interviewee proceeds to answer the second inquiry about their WTP. If the interviewee responds that they are willing to pay *B_i_* during the first inquiry, the second inquiry poses a higher WTP amount ($B_{i}^{U}$); conversely, if the interviewee is unwilling during the first round to pay *B_i_*, then the second inquiry amount ($B_{i}^{D}$) is lower. Thus, there are four possible responses for the interviewee’s decision-making behavior: (1) Responds willing for both inquiries; (2) responds unwilling for both inquiries; (3) responds willing in the first round and then unwilling in the second round; (4) responds unwilling in the first round and then willing in the second round. The probability of the derived combinations is, respectively, π^YY^, π^NN^, π^YN^, and π^NY^:

(6) $$\pi^{\mathrm{YY}}\left(B_{i},B_{i}^{U}\right)\;=\;\mathrm{Pr}\left\{B_{i}\;\leq {\;\mathrm{WTP}}_{\max}{\;\mathrm{and}\;B}_{i}^{U}\;\leq {\;\mathrm{WTP}}_{\max}\right\}\;=\;\mathrm{Pr}\left\{B_{i}\leq {\mathrm{WTP}}_{\max}\;|{\;B}_{i}^{U}\leq {\mathrm{WTP}}_{\max}\right\}\;\cdot \mathrm{Pr}\left\{B_{i}^{U}\leq {\mathrm{WTP}}_{\max}\right\}\;=\;\mathrm{Pr}\left\{B_{i}^{U}\leq {\mathrm{WTP}}_{\max}\right\}\;=\;1-G\left(B_{i}^{U};\;\theta \right)\;,$$

(7) $$\pi^{\mathrm{NN}}\left(B_{i},B_{i}^{D}\right)\;=\;\mathrm{Pr}\left\{B_{i}>WTP_{\max}{\;\mathrm{and}\;B}_{i}^{D}>{\mathrm{WTP}}_{\max}\right\}=G\left(B_{i}^{D};\;\theta \right),$$

(8) $$\pi^{\mathrm{YN}}\left(B_{i},B_{i}^{U}\right)\;=\;\mathrm{Pr}\left\{B_{i}\leq {\mathrm{WTP}}_{\max}\leq B_{i}^{U}\right\}\;=\;G\left(B_{i}^{U};\;\theta \right)-G\left(B_{i};\;\theta \right),$$

(9) $$\pi^{\mathrm{NY}}\left(B_{i},B_{i}^{D}\right)\;=\;\mathrm{Pr}\left\{B_{i}\geq {\mathrm{WTP}}_{\max}\geq B_{i}^{D}\right\}\;=\;G\left(B_{I};\;\theta \right)-G\left(B_{i}^{D};\;\theta \right).$$

Assuming interviewee (N) and assigned inquiry amounts ($B_{i},B_{i}^{U},B_{i}^{D}$), the log-likelihood function of interviewee i is:

(10) $${\mathrm{lnL}}^{D}\left(\theta \right)=\sum_{i=1}^{N}\left\{\begin{array}{c} d_{i}^{\mathrm{YY}}ln\pi^{\mathrm{YY}}\left(B_{i},B_{i}^{U}\right)+d_{i}^{\mathrm{NN}}ln\pi^{\mathrm{NN}}\left(B_{i},B_{i}^{D}\right)\\ +d_{i}^{\mathrm{YN}}ln\pi^{\mathrm{YN}}\left(B_{i},B_{i}^{U}\right)+d_{i}^{\mathrm{NY}}ln\pi^{\mathrm{NY}}\left(B_{i},B_{i}^{D}\right) \end{array}\right\}.$$

If the interviewee replies willing in both rounds, then $d_{i}^{\mathrm{YY}}=1$, $d_{i}^{\mathrm{NN}}=d_{i}^{\mathrm{YN}}=d_{i}^{\mathrm{NY}}=0$; conversely, if the interviewee replies unwilling in both rounds, then $d_{i}^{\mathrm{NN}}=1$, $d_{i}^{\mathrm{YY}}=d_{i}^{\mathrm{YN}}=d_{i}^{\mathrm{NY}}=\;$0; therefore, $d_{i}^{\mathrm{YN}}$ and $d_{i}^{\mathrm{NY}}$.

The first-order equilibrium solution ($\frac{\partial {\mathrm{lnL}}^{D}\left({\hat{\theta }}^{D}\right)}{\partial \theta }=0)\;$is taken from the log-likelihood function (${\mathrm{lnL}}^{D}\left({\hat{\theta }}^{D}\right)$) against the estimator (θ) to obtain the maximum approximate estimator ${\hat{\theta }}^{D}$. The asymptotic variance covariance matrix of ${\hat{\theta }}^{D}$ is expressed as:

(11) $$V^{D}\left({\hat{\theta }}^{D}\right)\;=\;{\left[-E\cdot \partial^{2}{\mathrm{lnL}}^{D}({\hat{\theta }}^{D})/\partial \theta \partial \;\theta \;\prime \right]}^{-1}\equiv I^{D}{\left({\hat{\theta }}^{D}\right)}^{-1},$$

where $I^{D}\left({\hat{\theta }}^{D}\right)$ represents the information matrix.

The relationship between the explanatory variable and WTP is represented as:

(12) $$\{\begin{array}{c} {\mathrm{WTP}}_{1i,\max}\;=\;X_{1i}^{\text{’}}\theta +\varepsilon_{1i}\\ {\mathrm{WTP}}_{2i,\max}\;=\;X_{2i}^{\text{’}}\theta +\varepsilon_{2i} \end{array}\;,$$

where $X_{i}^{\text{’}}$ is the exogenous variable vector, θ is the parameter vector, and $\varepsilon_{i}$ is the residual vector.

Haab and McConnell [37] posited that the original price and the second inquiry price set clear limits to the WTP or further limit the distribution where WTP is located. Cooper and Hanemann [38] argued that multiple statistical efficiency gains come from follow-up questions after the first inquiry and there is no need for the added complexity of a third inquiry. In addition, this study uses CVM as a measurement method and uses the bivariate probit model model by D-BDC method to estimate respondents WTP. For econometric estimation, a bivariate probit model was used for estimating the WTP [39], including environmental protection [40,41,42,43] and health [44,45,46].

### 2.3. Survey Design and Information

The survey used in this study was split into three parts. The first part introduced the study’s purpose and scope to the interviewees and inquired into their understanding of dengue fever and views on preventive behavior. The second part focused on the process of collecting information regarding WTP for dengue health risk prevention and control; this part explained the meaning and principle of extra payments and asked whether the interviewee was willing to pay the specified amount. The interviewer presented a chart to explain to the interviewees the current status of dengue fever in psychological health risks, illness-related health risks, and death risk. The interviewer used data to help the respondents understand the differences in the health risks of different types of dengue fever. According to the World Health Organization (WHO) and the Taiwan Centers for Disease Control (CDC), dengue fever symptoms include psychological factors, such as anxiety and depression, and even obvious physical symptoms, such as fever, headache, and soreness [47,48]. The most serious cases may cause shock and death. Without timely medical attention or treatment, the mortality rate can be as high as 20% [47]. Among them, the psychological health risks are divided into three types, fear, depression, and anxiety, allowing the respondents to understand different psychological aspects. The illness-related health risks are divided into headache, fever, soreness, and other symptoms, so that the respondents can understand the type of symptoms of illness. The death risk is death resulting from dengue disease. The third part relates to the collection of information about the interviewee, such as their socioeconomic background, age, income level, gender, and level of education.

The pretest survey posited a scenario in which the government has established a dengue prevention institution that is financed through a trust fund and used open inquiry to collect the interviewee’s WTP to lower psychological and illness-related health risks and death risk through preventive actions such as removing dengue fever breeding sources, case control, and regulations. According to Alberini’s method [39], the interviewee’s WTP was arranged from lowest to highest, and 10% of the payment amount was eliminated from the lowest and highest as an observational error. Subsequently, the 20th, 40th, 60th, and 80th quantile of the remaining WTP were taken as the basis for the first round of WTP inquiries. If the interviewee was willing to pay the amount in the first inquiry, then the payment amount in the second inquiry was doubled; if the interviewee was unwilling to pay the amount in the first inquiry, then the amount in the second inquiry was halved. The participants of this study were divided into groups A, B, C, and D; the inquiry payment amounts are listed in Table 1.

**Figure 1:**
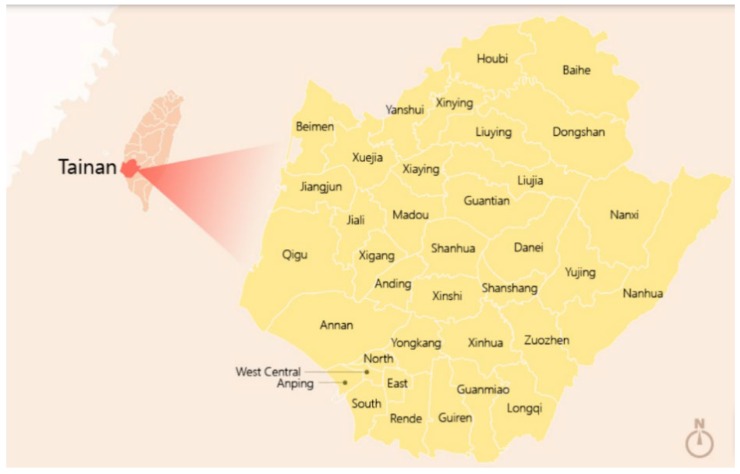
Location of survey site in Tainan. Data source: Tourism Bureau of Tainan City Government [49].

In this study, stratified sampling was used for data collection. Stratified sampling is a probability sampling method and a form of random sampling in which the population is divided into two or more groups. The application of the stratified sampling method involves dividing the population into different subgroups and selecting subjects from each group in a proportionate manner. Therefore, this study conducted stratified sampling of the residents of Tainan City, with a total of 37 administrative districts (Figure 1), by distributing the survey in proportion to the population of each administrative district, with interviewers conducting one-on-one interviews. It is based on the government’s announcement of the population of various regions of Tainan City in 2017, and the sampling is based on the proportion of the population. The number of sample distributions by region is as Table 2.

This study was approved by the Human Research Ethics Review Committee of National Cheng Kung University in Taiwan before conducting the questionnaire (Review Number: NCHU HREC-E-105-131-2) and only to evaluate the public’s behavior of dengue prevention. Most of the study participants did not suffer ethical harm. If the respondents had negative feelings, the interview was terminated. To ensure the quality of the data collected by face-to-face interviews and to explain the objectives of the survey, we recruited five investigators who were trained in investigative skills. Interviewees were willing to accept the survey for this study with anonymity, and confidentiality was assured. The questionnaire does not involve personal privacy information, including name, identity card number, date of birth, address or telephone number, and it is a non-registered questionnaire. A total of 791 surveys were distributed, and after eliminating 61 invalid responses, a total of 730 valid responses were analyzed.

## 3. Results

### 3.1. Demographics

Table 3 shows that 44.11% of the interviewees were men and 55.89% were women. About 59.86% of interviewees were between the ages of 30 and 49. Married interviewees were 53.29% of the interviewees, and single interviewees were 46.71%. Interviewees with children were 47.12% of the sample. Nearly half of the respondents (49.18%) held a bachelor’s degree, and 15.2% held a master’s degree or above. The majority average monthly income was between NTD$20,000 and NTD$80,000 and was 70.82% of all samples. Moreover, 13.01% of the participants responded affirmatively that there had been a confirmed case of dengue fever in the past near their home.

### 3.2. Dengue Fever Perceptions and Preventive Behaviors

Table 4 presents results of the analysis of the public’s perception of dengue fever. The study results indicate that 65% of interviewees understood that the epidemic is transmitted by the *Ae. aegypti* mosquito, but 61% of interviewees were unaware that, currently, there is no vaccine against dengue. Regarding preventive behaviors, most interviewees believed that removing vector mosquito breeding sources is the most effective method of eradicating dengue infection, but only 17% of interviewees reported participating in neighborhood cleanup and prevention activities, demonstrating a lack of public action. In terms of preventive behavior cognition, the awareness of removing vector mosquitoes is the most important way to remove dengue fever. An interval scale of 1 to 4 indicates that more than 50% of the respondents scored 3~4 points, followed by 4~5 points.

### 3.3. Estimation Results

This study used the D-BDC format with hypothetical CVM scenarios to survey the public about its WTP for dengue prevention to lower health risks. The variables of this study were mainly selected based on the literature, home interviews, and the characteristics of dengue infection. In terms of demographics variables, income is one of the commonly selected variables and one of the important indicator variables for WTP measurement. Tseng et al. [50] stated that the income variable is one of the important indicators of WTP estimation, and incorporating income into one of the variables to evaluate the public’s WTP for dengue prevention. In addition, high household income has a significant relationship to the onset or mortality of the disease and reduces concerns about health risks [51]. It is worth mentioning that there may be a problem of collinearity between the education and income variables in the WTP model. Hence, this study examined the variance inflation factor (VIF) value if educational variables were included in the model, and the result shows that the VIF value of the education variable is 11.52. Stewart [52] pointed out when the VIF value of the measurement variable is greater than 10, there is a collinearity problem in the model evaluation. In addition, according to WTP theory, income is an important basis for assessing an individual’s willingness to pay. Therefore, this study selects income variable as one of the important measurement variables.

Area variables are mainly areas where dengue fever has occurred, and it is even more obvious that residents attach great importance to the prevention of dengue fever. Whether in the area where the disease occurs or in the neighbourhood area, people are indirectly alert to the disease [5]. In terms of gender, men and women have different sensitivities to disease prevention. Chuang et al. [5] found that a relatively high proportion of women are infected with dengue fever. Nielsen et al. [51] also pointed out that gender is significant in health risk assessment and has been found to potentially affect the reduction of health risks. Further, families with children have more sensitivity to disease prevention. Children are also found to be the main group infected with dengue fever [5]. Regarding the disease prevention variables of dengue fever, disease awareness (infection vector, know vaccine) and prevention behaviors (activity, cognition) are often among the important indicators of disease prevention. Bota et al. [53] explored the impact of the public on knowledge, cognition, and attitudes related to dengue fever. Among them, it was found that the respondents’ knowledge and cognition of dengue fever vectors were significant. Some countries have strengthened the awareness and prevention of community residents with dengue fever [10,11].

To sum up, the WTP model for participating in dengue prevention is:

ln WTP = f (Gender, Income, Children, Area, Infection vector, Know vaccine, Activity, Cognition),(13)

where ln WTP is the WTP for dengue fever prevention. Gender is a virtual parameter that represents the interviewees’ gender (male = 1, female = 0), and income is the interviewees’ average monthly income. Children is a virtual parameter, representing whether there are children under the age of 18 in the household (0 = no, 1 = yes). Area is also a virtual parameter that represents whether a dengue epidemic has ever occurred in the interviewee’s area of residence (0 = no, 1 = yes). Regarding dengue fever perceptions and behaviors, the infection vector virtual parameter refers to the understanding that the dengue infection vector is the *Ae. aegypti* mosquito (0 = no, 1 = yes), and the know vaccine virtual parameter is the understanding that there is currently no dengue vaccine (0 = no, 1 = yes). Activity is the virtual parameter for having participated in neighborhood cleanup and prevention activities (0 = no, 1 = yes), and cognition is the level of awareness that removing vector mosquito breeding sources eradicates dengue infection (from 1 to 5). After building an assessment model based on the aforementioned information, the model was run through empirical model evaluation using Weibull probability distribution and maximum likelihood estimation.

As shown in Table 5, the income variable in WTP for psychological and illness-related health risks and death risk was uniformly positive and significant, suggesting that interviewees with higher incomes have higher WTP for dengue prevention. The area variable was uniformly positive and significant across psychological and illness-related health risks and death risk WTPs, suggesting that interviewees who live in areas that were once epidemic zones have higher WTP for dengue prevention. The gender variable in psychological health risk WTP is negative and significant, suggesting that women’s WTP for dengue prevention is higher than men’s. However, gender is not significant for death risk, which means that both men and women may be important to the health risk of death, and there may not be a clear evaluation of the differences in the risk of death from gender variables. Studies have shown that exploring the incidence or mortality of dengue fever has no statistical significance with gender [5]. In assessing factors that reduce health risks, gender variables are significant, and the results show that women are relatively more health conscious [51]. Uniformly positive and significant children variables for illness-related health risk and death risk WTP suggest that families with children have higher WTP for dengue prevention. However, the children variable is not significant for psychological risk factors, the main reason may be that Tainan was the region with the most severe dengue fever in Taiwan, and each respondent, whether or not they had children, might have greater psychological anxiety towards dengue fever which may not have significant differences. In most dengue endemic countries in Southeast Asia and Latin America, children are typically considered the most at-risk group, especially in morbidity or mortality risk having significance [54,55,56].

In regard to dengue fever perceptions and behavior, the dengue fever infection vector variable was not significant across the three health risk functions although the infection vector is one of the most important indicators of disease prevention. The probable factor is the time when data were collected in this study; approximately 65% of respondents knew the infection vector of dengue fever, and there is relatively little variability in the infection vector variable, which indirectly leads to insignificant results. According to the WHO, dengue fever infectious disease is mainly transmitted via *Ae. aegypti* mosquito, and a clean living environment has become the main way of preventing dengue fever [47]. Therefore, households’ awareness of dengue fever vectors indirectly increases environmental cleanliness [57,58]. People can always pay attention to the cleanliness of environmental health, which can help slow down the breeding of mosquitoes [59]. The no vaccine variable had a positive and significant influence on WTP in illness-related health risk and death risk, suggesting that interviewees who understand vaccine prevention have higher WTP. The variable for having participated in neighborhood cleanup prevention activities (Activity) was uniformly positive and significant in WTP for illness-related health risk and death risk, demonstrating that interviewees who have participated in dengue prevention activities have higher WTP.

Using the estimations in Table 5, this study further estimated the interviewees’ WTP for different health risks. According to the estimation result of the variable coefficient value in Table 5, it is necessary to substitute the mathematical Formula (5) for the estimation. Each respondent‘s WTP in psychology, illness, and death health risks can be estimated, and the WTP of all respondents to evaluate the final WTP value for the health risks of dengue fever prevention can be summarized. The results are shown in Table 6. Across 730 interviewees facing psychological and illness-related health risks and death risk, each person was willing to pay NT$751 on average each year to prevent psychological health risks, with a confidence interval of NT$688.26–NT$824.14; each person was willing to pay NTD$793 on average each year to prevent illness-related health risks, with a confidence interval of NT$746.04–$847.83; and each person was willing to pay NT$1086 on average each year to prevent death risk, with a confidence interval of NT$1021.29–NT$1143.69.

## 4. Discussion

This study used CVM to build a WTP model regarding dengue prevention and control and assessed WTP efficacy when affected by psychological, illness-related, and death-related health risks through questionnaire surveys. The primary purpose was to evaluate the effects of dengue prevention and control on lowering the threat of infection or death by an epidemic on residents. The study results are presented below.

### 4.1. Socioeconomic Background Factors

Men and women exhibited a significant difference in perceptions and behavior activities with respect to dengue prevention and control. For the most part, women take on the role of managing household affairs and taking care of the family; thus, especially in the face of a high risk of epidemics, they are sensitive to the home environment. Because of the heightened psychological stress during epidemics, women end up bearing a higher psychological cost compared to men. The effect of income on the health risk appraisal model—whether affected by psychology, illness, or death—was positive and significant, suggesting that the income variable plays a major role in explaining the model assumptions. This result is consistent with the empirical findings of Wang et al. [60] and with the hypothesis that the demand for health benefits should increase with income, as proposed by Freeman [61] in his environmental economics theory. Therefore, this study estimated that high-income earners would be more willing to pay to lower health risks. Families with members younger than 18 years old are typically more sensitive to the risk of epidemic diseases, especially those that threaten illness or death; therefore, they are willing to invest higher amounts than families without children during dengue epidemics, especially to lessen the risk of sickness and death. Residents of neighborhoods where outbreaks have occurred are willing to invest higher amounts in dengue prevention to reduce health risks than residents of other neighborhoods. A major contributor to this phenomenon is a severe outbreak in Tainan City, Taiwan in 2015, which resulted in a considerably heightened emphasis on cleanliness in the home environment to mitigate the psychological and illness-related health risks and risk of death.

### 4.2. Public Perceptions and Behaviors in Prevention and Control

Knowledge of the absence of a dengue vaccine and the effective means of prevention can lead to greater insight into residents’ understanding of dengue prevention. Presently, an efficacious dengue vaccine is lacking; therefore, residents who knew about dengue epidemic prevention were willing to invest more in prevention activities than other residents—this effect becomes more pronounced when facing the risk of illness or death. Residents who have participated in dengue prevention and cleanup activities were willing to invest in higher prevention expenditure, regardless of the type of health risk. As such, awareness campaigns and prevention measures initiated in advance—seminars, advocacy meetings, and similar efforts—become key factors in containing an outbreak. Moreover, people with higher levels of awareness were willing to invest more in dengue prevention.

## 5. Conclusions

The prevention of a dengue epidemic relies on the day-to-day cleanliness of people’s home environments and the eradication of the breeding sites of vector mosquitoes. We surveyed the public to determine their WTP to lower the health risks related to dengue fever and how their perceptions of health risks affect that WTP. The research demonstrated that in dengue prevention and control, the cleanliness of the surrounding environment is a crucial preventive behavior for each household. During an outbreak, the asymmetrical spread of information can lead to psychological effects in the public, such as fear, anxiety, and depression. As a result, the government needs to disseminate comprehensive information regarding epidemic prevention as well as undertake effective physical actions focusing on vector mosquito control. In regard to implementation and management, the government should regularly hold dengue prevention activities every year, regardless of the season, so that residents can develop critical habits and receive correct knowledge regarding dengue prevention.

## Figures and Tables

**Table 1:** Double-bounded dichotomous choice; payment and inquiry amount.

Starting Price (Survey Groups)	Willingness to Pay Each Year		
	Psychological Health Risks	Illness-Related Health Risks	Death Risk
Group A	100 (50/200)	100 (50/200)	150 (75/300)
Group B	200 (100/400)	300 (150/600)	400 (200/800)
Group C	500 (250/1000)	700 (350/1400)	1000 (500/2000)
Group D	1000 (500/2000)	1000(500/2000)	1100 (550/2200)

Notes: (1). Nonparenthesized number is the first inquiry amount, and the parenthesized number is the second inquiry amount. The second inquiry amount is determined by the first response. If the first response is *willing*, the second inquiry price is raised; if the first response is *unwilling*, then the second inquiry price is lowered. (2). The unit price is NTD, and the exchange rate is US$1 = NT$31.

**Table 2:** Number of sample distributions by region.

District	Population (%)	Sample	District	Population (%)	Sample	District	Population (%)	Sample
North	7.04	56	Xinhua	2.32	18	Xinying	4.14	33
East	9.96	79	Anding	1.61	13	Liuying	1.13	9
South	6.67	53	Shanhua	2.54	20	Guantian	1.14	9
Anping	3.49	28	Xigang	1.32	10	Liujia	1.19	9
Annan	10.16	80	Madou	2.37	19	Houbi	1.27	10
Yongkang	12.34	98	Jiali	3.15	25	Baihe	1.52	12
Rende	3.98	31	Qigu	1.23	10	Dongshan	1.12	9
Guiren	3.62	29	Jiangjun	1.06	8	Danei	0.53	4
Guanmiao	1.83	14	Beimen	0.60	5	Shanshang	0.39	3
Longqi	0.22	2	Xuejia	1.39	9	Nanxi	0.52	4
Xinshi	1.93	15	Yanshui	1.37	11	Nanhua	0.47	4
Yujing	0.75	6	Zuozhen	0.26	2	Total	100	791
West Central	4.09	32	Xiaying	1.29	10			

**Table 3:** Demographic characteristics of respondents.

Variable		N	%	Variable		N	%
Gender	Male	322	44.11	Monthly Income (NTD)	Below 20,000	63	8.63
	Female	408	55.89		20,000–40,000	193	26.44
Age	<20	10	1.37		40,000–60,000	179	24.52
	20–29	161	22.05		60,000–80,000	145	19.86
	30–39	202	27.67		80,000–100,000	68	9.32
	40–49	235	32.19		100,000–150,000	60	8.22
	50–59	101	13.84		Above 150,000	22	3.01
	60s or over	21	2.88	Children	Child	344	47.12
Marital Status	Married	389	53.29		No Child	386	52.88
	Single	341	46.71	Confirmed Case Near Where They Live	Yes	95	13.01
Education Degree	Below high school	260	35.62		No	635	86.99
	Bachelor’s	359	49.18	Total		730	100
	master’s or above	111	15.20				

Note: The unit price is NTD$, and the exchange rate is US$1 = NTD$31.

**Table 4:** Statistics of dengue perception.

Variable	Definitions		N	%
Infection vector awareness	Virtual parameter, understanding the *Ae. aegypti* mosquito is the dengue infection vector (0 = no; 1 = yes)	0	255	34.93
		1	475	65.07
Vaccine prevention awareness	Virtual parameter, understanding there is currently no vaccine to prevent dengue (0 = no; 1 = yes)	0	445	60.96
		1	285	39.04
Preventive behavior and activities	Virtual parameter, participating in neighborhood cleanup preventive activities (0 = no; 1 = yes)	0	606	83.01
		1	124	16.99
Preventive behavior awareness	Level of awareness that removing vector mosquito breeding sources eradicates dengue infections (from 1 to 5)	1~2	7	0.96
		2~3	24	3.29
		3~4	395	54.11
		4~5	304	41.64

**Table 5:** Function estimation results for health risks and willingness to pay (WTP) for dengue prevention (Weibull distribution).

Variables	Coefficient Estimates (*t*-Values)		
	Psychological Health Risks	Illness-Related Health Risks	Death Risk
Constant	5.81	3.59	4.99
	(16.71) **	(12.66) **	(15.14) **
Gender	−1.46 × 101	−2.39 × 101	−2.67 × 101
	(−3.92) *	(−4.05)	(−4.21)
Income	5.91 × 106	6.26 × 106	6.82 × 106
	(0.72) **	(0.86) ***	(0.91) **
Children	3.81 × 101	3.15 × 101	4.27 × 101
	(4.61)	(4.32) *	(4.56) **
Area	2.67 × 101	1.59 × 101	2.91 × 101
	(1.43) **	(1.07) ***	(1.48) **
Infection vector	2.18 × 101	3.61 × 101	2.66 × 101
	(1.23)	(2.34)	(1.41)
Know Vaccine	1.58 × 101	1.82 × 101	2.84 × 101
	(1.14)	(1.43) *	(1.54) **
Activity	1.46 × 101	2.61 × 101	2.06 × 101
	(1.55) *	(1.94) **	(1.35) **
Cognition	3.71 × 101	5.63 × 101	5.68 × 101
	(7.49)	(8.72) *	(8.77) **
Scale	6.87 × 101	8.69 × 101	8.31E-01
	(30.19) **	(32.94) **	(31.22) ***
Log likelihood	−476.33	−522.76	−512.62
Log likelihood ratio	42.18 ***	44.26 ***	43.85 ***

Note: (1). *, **, *** respectively show significance under 10%, 5%, 1%. (2). Log likelihood ratio = (−2) × (Restricted Log likelihood–Log likelihood), X2 (8,0.01) = 20.09.

**Table 6:** Health risks and WTP for dengue prevention.

Appraised Health Risk	WTP/Person/Year	95% Confidence Interval
Psychological	751	(688.26–824.14)
Illness-related	793	(746.04–847.83)
Death	1086	(1021.29–1143.69)

Note: unit price is NT$, and exchange rate is approximately US$1 = NT$31.
